# Editorial: Artificial intelligence-assisted medical imaging solutions for integrating pathology and radiology automated systems, volume II

**DOI:** 10.3389/fmed.2025.1686859

**Published:** 2025-09-18

**Authors:** Prabhishek Singh, Vinayakumar Ravi, Anchit Bijalwan

**Affiliations:** ^1^School of Computer Science Engineering and Technology, Bennett University, Greater Noida, India; ^2^Center for Artificial Intelligence, Prince Mohammad bin Fahd University, Khobar, Saudi Arabia; ^3^School of Computing and Innovative Technologies, British University Vietnam, Hanoi, Vietnam

**Keywords:** pathology, radiology, computer-aided diagnosis, explainable AI, generalized models, volume II

## 1 Introduction

This Research Topic, *Artificial Intelligence-Assisted Medical Imaging Solutions for Integrating Pathology and Radiology Automated Systems - Volume II*, has successfully published 21 high-quality articles, reflecting the growing interest and advancements in this domain. The primary objective of this collection is to identify the fundamental research challenges at the intersection of pathology and radiology, particularly considering the increasing integration of Artificial Intelligence (AI) technologies. With AI becoming instrumental in enhancing diagnostic accuracy and efficiency, there is a pressing need to understand its real-world applicability across medical modalities and whole-slide imaging. This volume emphasizes the development of AI-driven systems that unify diagnostic inputs—textual, visual, and molecular—to support precise clinical decision-making. By fostering interdisciplinary collaboration, this Research Topic provides valuable insights into how AI can transform both translational and clinical research, ultimately contributing to improved patient outcomes in pathology and radiology practices.

## 2 Contributions

A total of 21 publications have been published on this Research Topic. Xie et al. proposed a blockchain-based swarm learning framework for orthopedic fracture diagnosis, enabling decentralized, privacy-preserving collaboration across institutions. Their model achieved expert-level accuracy without centralizing patient data. He et al. proposed a deep learning-based framework using an optimized Res-UNET with deep supervision for integrating pathology and radiology, enabling accurate tumor segmentation and cross-modal diagnosis. Maqsood et al. proposed an optimized Res-UNET architecture enhanced with deep supervision for brain tumor segmentation in MRI scans. Their model demonstrated significantly improved accuracy over traditional U-Net approaches, highlighting its potential for clinical neuro-imaging applications. Rani et al. introduced hybrid SMOTE-ENN data balancing with ensemble models (KNN and AdaBoost) for enhancing liver disease diagnosis on real-world patient datasets. Their approach effectively addressed class imbalance and significantly improved predictive accuracy over traditional classifiers. Zhang et al. proposed DualDistill, a dual-guided self-distillation framework combining intra-frame relationship modeling and spatial-temporal attention mechanisms to enhance deep learning classification of carotid plaque in ultrasound videos. Applied across 13 model architectures on 317 clinical cases, it boosted accuracy by an average of 2.97%, peaking at 4.74%, without increasing inference complexity. Sun introduced MedFusion-TransNet, a transformer-based framework that fuses multi-modal medical imaging using hierarchical feature encoding and attention-driven dynamic optimization (CASNet + DRGO), significantly boosting segmentation accuracy and boundary precision across benchmark datasets. Baili et al. proposed two deep learning models—RbACNN and IRbACNN—enhanced with attention mechanisms and explainable-AI techniques to classify early-stage Alzheimer's and Parkinson's disease from medical imaging. Their framework achieved improved accuracy and interpretability for multi-class neurodegenerative diagnosis.

Amin et al. proposed a boundary-aware segmentation network (BASNet) that pairs multiscale U-Net-style prediction with a residual refinement module and hybrid loss to improve skin-lesion segmentation under challenging imaging conditions. Al-Adhaileh et al. proposed a transfer learning framework employing MobileNetV2, VGG-16, and ResNet50V2 for categorizing pediatric chest X-ray images into pneumonia and normal classes. Alarjani and Almarri proposed an fMRI-based framework employing Multivariate Pattern Analysis (MVPA) for feature extraction and an Extreme Learning Machine (ELM) classifier to distinguish Alzheimer's disease stages (NC, MCI, AD). Their model achieved up to 94.0% accuracy in multi-class classification across ADNI and OASIS datasets, demonstrating robust, efficient functional connectivity–based diagnosis. Aldhyani et al. developed a deep learning framework using transfer learning with VGG16, ResNet152V2, and DenseNet201—enhanced via attention mechanisms and dilated convolutions—for automated bone fracture detection on a 10,580-image X-ray dataset. DenseNet201 emerged as the top performer, achieving ~97% validation accuracy, outperforming the other architectures. Ding et al. evaluated GPT-4's diagnostic accuracy on 144 histopathology images from various organs and colorectal biopsies, comparing its performance to that of pathology residents. GPT-4 achieved an overall accuracy of ~64% in tissue origin identification and 75%−88% for classifying dysplasia and adenocarcinoma, showing sensitivity on par with pathology trainees. Buttar et al. proposed a specialized deep convolutional neural network (DCNN) for automated detection and classification of neurological disorders—including Alzheimer's, Parkinson's, and epilepsy—from MRI scans. The model achieved a classification accuracy of 98.44%, outperforming benchmarks like ResNet-50 and AlexNet with superior precision, recall, and F1-scores. Maity et al. introduced a deep learning framework combining Deep-Residual U-Net and DeepLabV3+ for precise hippocampus and ventricle segmentation in fMRI data, followed by classification using hybrid models like VGG-16-RF and VGG-16-SVM for early Alzheimer's diagnosis.

Alharbi et al. proposed a deep feature-extraction–based framework combining four CNN variants (original, no-batch-norm, few-filters, and strided) and hybrid models (CNN-SVM, CNN-RF, and CNN-LR) for multi-class dermoscopic skin cancer classification. Alenezi et al. presented a Multiscale Attention-over-Attention Network for retinal disease classification using OCT images, integrating EfficientNetB7 backbone with pyramidal attention, channel and spatial refinement mechanisms to capture both global and local retinal features. Sathya et al. proposed an enhanced Xception-based convolutional neural network—augmented with batch normalization, dropout layers, and transfer learning—to classify meningioma, glioma, and pituitary tumors from brain MRI scans. The model achieved 98.04% accuracy, with precision and recall above 96%, demonstrating robust generalizability across tumor types and reliable clinical diagnostic potential. Rauf et al. introduced two custom deep-learning architectures (3- and 4-residual block CNNs) optimized with Bayesian hyperparameter tuning and an improved Generalized Normal Distribution Optimizer for fetal ultrasound classification. Almarri et al. introduced GANVesselNet, a dual-path GAN-based framework for retinal vessel segmentation in fundus images, integrating an autoencoder-decoder and a UNet-inspired branch to capture multi-scale vessel structures. On STARE and DRIVE datasets, it attained standout performance—sensitivity ~81.74%, specificity ~98.62%, and accuracy ~98.27% on STARE, and similarly high metrics on DRIVE—exceeding traditional segmentation methods. Alzakari et al. introduced LesionNet, a skin lesion classification system that combines Scale-Invariant Feature Transform (SIFT) features with a custom convolutional neural network. Evaluated on the HAM10000 dataset, the model achieved an outstanding 99.28% classification accuracy, demonstrating exceptional robustness against typical imaging artifacts like hair, low contrast, and ruler markers. Alqhatni et al. present an EfficientNet-B0-based deep learning model, enhanced with Grad-CAM, for precise lung cancer stage classification from CT images. They demonstrate high accuracy and interpretability, highlighting its potential for reliable and transparent automated diagnosis.

## 3 Conclusion

This Volume-II of editorial presents 21 research papers exploring AI-assisted medical imaging for integrating automated pathology and radiology systems. The aim was to curate impactful work within AI-driven healthcare. The contributions reflect a growing trend toward intelligent diagnostic solutions, with future efforts encouraged to further enhance integration across both domains using advanced AI techniques.

